# Supplementary material for: The endothelial-enriched lncRNA LINC00607 mediates angiogenic function
Source: Basic Res Cardiol. 2023 Jan 26;118(1):5. doi: 10.1007/s00395-023-00978-3 (PMC9879848; doi:10.1007/s00395-023-00978-3)
Supplement: Supplementary file 1 — Supplementary file1 (PDF 9341 KB) [file 395_2023_978_MOESM1_ESM.pdf]

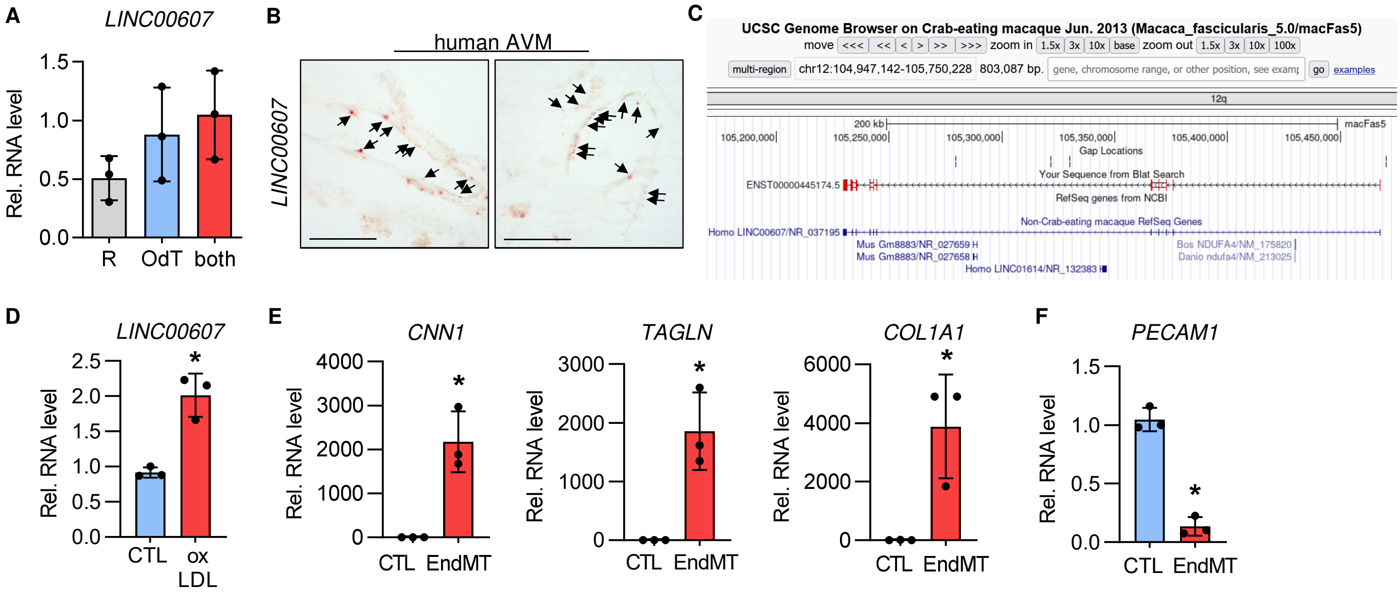

Sup. Fig. 1:

A, Relative RNA expression of *LINC00607* in HUVEC after RT-qPCR with either only random hexamer primers (R), only Oligo(dT) primers (OdT) or the combination of both (both). n=3, Paired t-test. **B**, RNA in situ hybridization of human arteriovenous malformations (AVM) sections with RNAscope. Arrows point to dots indicating *LINC00607* RNA. Representative images from 2 different patient samples are shown. Scale bar indicates 20 µm. **C**, UCSC Genome browser view of the *LINC00607* homologue in *Macaca fascicularis*. BLAT nucleotide sequence alignments of cDNA of human versus *Macaca fascicularis* are shown in red. **D**, Relative LINC00607 expression in HUVEC treated with oxLDL (16h, 10 µg/mL). n=3, Unpaired t-test. **E**, Relative RNA expression of EndMT control genes Calponin 1 (CNN1), Transgelin (TAGLN) and Collagen, type I, alpha 1 (COL1A1) in HUVEC under basal (CTL) or Endothelial-to-mesenchymal transition (EndMT) conditions. n=3, Unpaired t-test. **F**, Relative RNA expression of endothelial control gene Platelet endothelial cell adhesion molecule-1 (PECAM1) in HUVEC under basal (CTL) or EndMT conditions. n=3, Unpaired t-test. Error bars are defined as mean +/- SD. *p<0.05.

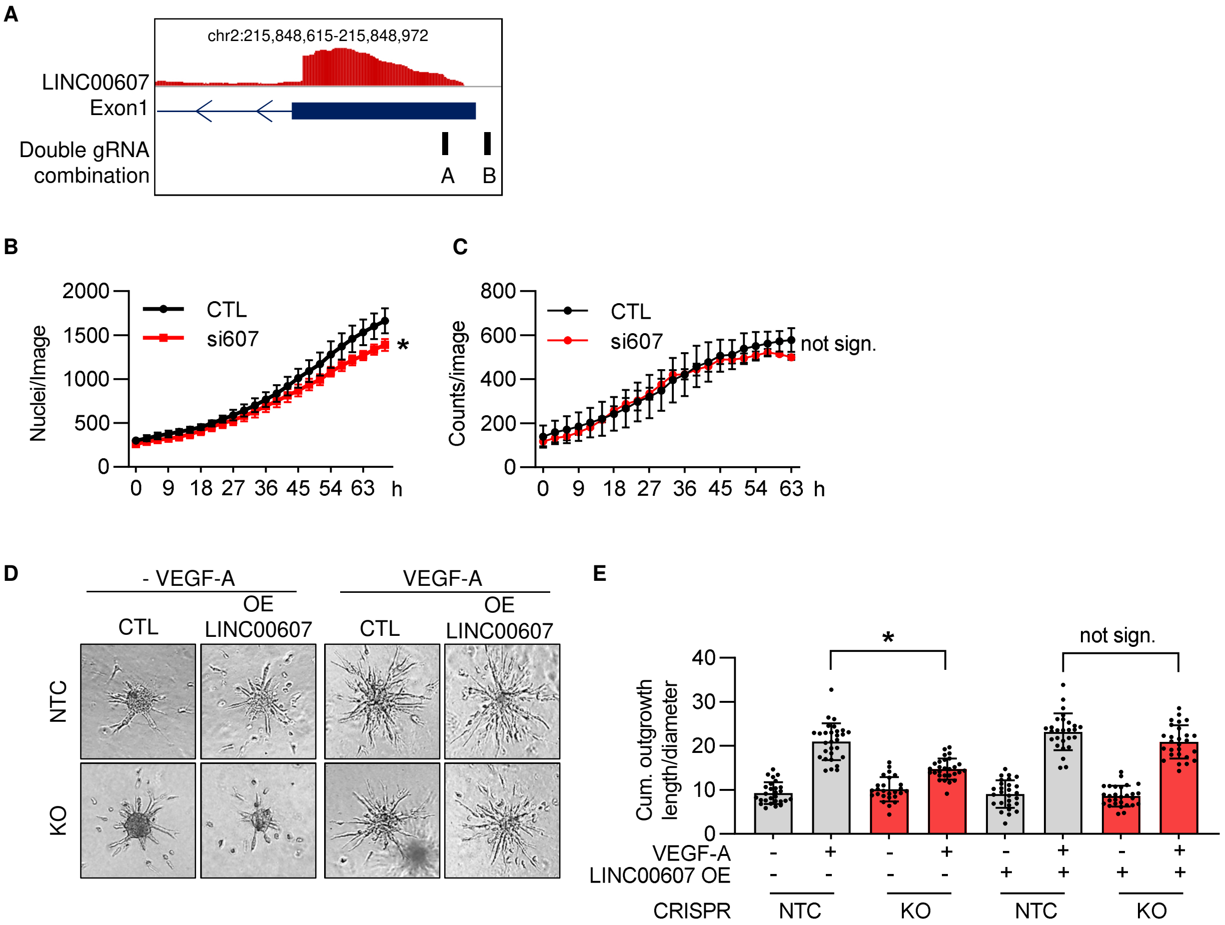

Sup. Fig. 2:
A, Scheme of gRNAs used for CRISPR/Cas9-mediated KO of LINC00607. **B**, Proliferation assay of HUVEC with siRNA-mediated LINC00607 knockdown. Nuclear count per image per well. 5000 cells/well of a 96-well plate and pictures taken every 3 h. siRNA knockdown was performed 16 h prior to seeding for proliferation. n=3, Area under the Curve followed by unpaired t-test. **C**, Apoptosis assay of HUVEC with siRNA-mediated LINC00607 knockdown. Apoptotic cell count per image per well. Cells seeded at a density of 7500 cells/well of a 96-well plate and pictures taken every 3 h. n=3, Area under the Curve followed by unpaired t-test. **D**, Spheroid outgrowth assay of HUVEC after CRISPR/Cas9-mediated LINC00607 KO or non target control (NTC). Cells treated with and without VEGF-A conditions for 16 h with/ without LINC00607 overexpression (OE) are shown. Empty vector transfection served as control (CTL). **E**, Quantification of the ratio of cumulative outgrowth length and respective spheroid diameter from spheroid outgrowth assay. n=26-29, Three-way ANOVA with Bonferroni post hoc test. Error bars are defined as mean +/- SD. *p<0.05.

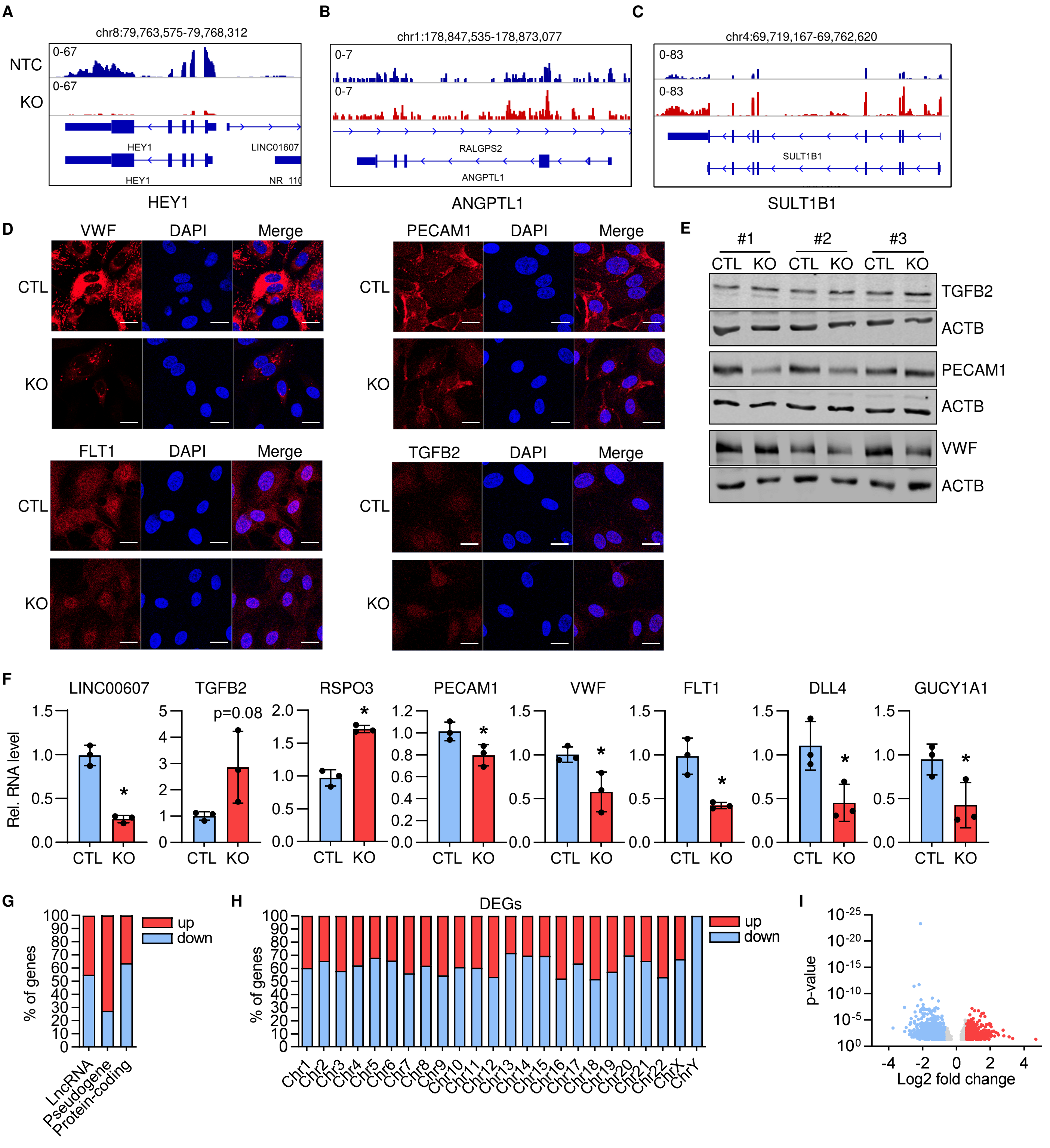

Sup. Fig. 3:
A-C, Examples of significantly down- and up-regulated genes after *LINC00607* KO. IGV genome tracks of the *HEY1*, *ANGPTL1* and *SULT1B1* locus. Shown are genomic tracks of *LINC00607* KO (red) and NTC (blue); tracks of three replicates are overlaid. **D,** Immunofluorescence with antibodies against VWF (Abcam, ab6994), PECAM1 (Santa Cruz, sc-376764), FLT1 (ThermoFisher, #36110) and TGFB2 (Santa Cruz, sc374658) in HUVEC with (KO) or without (NTC) CRISPR/Cas9-mediated knockout of LINC00607. Nuclei were stained with DAPI. Scale bar indicates 20 µm. **E**, Western analysis with antibodies against VWF, PECAM1, TGFB2 and β-Actin of control (NTC) or LINC00607 KO HUVEC. Three different batches of HUVEC were used. **F,** RT-qPCR of differentially expressed genes after LINC00607 KO: LINC00607, Transforming growth factor beta 2 (TGFB2), R-Spondin 3 (RSPO3), Platelet endothelial cell adhesion molecule-1 (PECAM1), von Willebrand Factor (VWF), Fms Related Receptor Tyrosine Kinase 1 (FLT1), Delta Like Canonical Notch Ligand 4 (DLL4), Guanylate Cyclase 1 Soluble Subunit Alpha 1 (GUCY1A1) n=3, Unpaired t-test. **G-H,** Percentage of genes belonging to lncRNAs, pseudogenes or protein-coding genes (G) or chromosomal distribution and percentage of genes (E) up- or downregulated (DEGs) in the RNA-Seq after *LINC00607* KO in HUVEC. Only genes with a log2 fold change of +/-0.585, a basemean expression of 5 and a p-adjusted value <0.05 are shown. **I**, Volcano plot of ATAC-Seq showing the log2 fold change (KO vs. NTC) of all peaks against their p-value. Error bars are defined as mean +/- SD. *p<0.05. Genomic coordinates correspond to hg38.
