# Supplementary material for: The endothelial-enriched lncRNA LINC00607 mediates angiogenic function
Source: Basic Res Cardiol. 2023 Jan 26;118(1):5. doi: 10.1007/s00395-023-00978-3 (PMC9879848; doi:10.1007/s00395-023-00978-3)
Supplement: Supplementary file 2 — Supplementary file2 (DOCX 18 KB) [file 395_2023_978_MOESM2_ESM.docx]

**Data Deposition Information Item**

**The endothelial-enriched lncRNA LINC00607 mediates angiogenic function**

Frederike Boos^1,2^, James A. Oo, Timothy Warwick, Stefan Günther, Judit Izquierdo Ponce, Melina Lopez, Diba Rafii, Giulia Buchmann, Minh Duc Pham, Zahraa S. Msheik, Tianfu Li, Sandra Seredinski, Shaza Haydar, Sepide Kashefiolasl, Karl H. Plate, Rüdiger Behr, Matthias Mietsch, Jaya Krishnan, Soni S. Pullamsetti, Sofia-Iris Bibli, Rabea Hinkel, Andrew H. Baker, Reinier A. Boon, Marcel H. Schulz, Ilka Wittig, Francis J. Miller, Ralf P. Brandes^1,2^* and Matthias S. Leisegang^1,2^*

^1^Institute for Cardiovascular Physiology, Goethe University, Frankfurt, Germany

^2^German Center of Cardiovascular Research (DZHK), Partner site RheinMain, Frankfurt, Germany

* shared senior authors

Correspondence to:

Matthias S. Leisegang, PhD or Ralf P. Brandes, MD

Institut für Kardiovaskuläre Physiologie

Fachbereich Medizin der Goethe-Universität

Theodor-Stern-Kai 7

60590 Frankfurt am Main, Germany

Tel.: +49-69-6301-6996

Fax.: +49-69-6301-7668

Email: [Leisegang@vrc.uni-frankfurt.de](mailto:Leisegang@vrc.uni-frankfurt.de)

Email: [Brandes@vrc.uni-frankfurt.de](mailto:Brandes@vrc.uni-frankfurt.de)

*Data availability*

The RNA-Seq and ATAC-Seq datasets have been deposited and are available at NCBI GEO with the accession number GSE199878:

<https://www.ncbi.nlm.nih.gov/geo/query/acc.cgi?acc=GSE199878>

BRG1 CUT&RUN datasets have been deposited and are available at NCBI GEO with the accession number GSE201824:

<https://www.ncbi.nlm.nih.gov/geo/query/acc.cgi?acc=GSE201824>
